# Dystrophin Dp116: A Yet to Be Investigated Product of the Duchenne Muscular Dystrophy Gene

**DOI:** 10.3390/genes8100251

**Published:** 2017-10-02

**Authors:** Masafumi Matsuo, Hiroyuki Awano, Masaaki Matsumoto, Masashi Nagai, Tatsuya Kawaguchi, Zhujun Zhang, Hisahide Nishio

**Affiliations:** 1 Department of Physical Therapy, Faculty of Rehabilitation, Kobe Gakuin University, Kobe 651-2180, Japan; zhang_zhujun@hotmail.com; 2 Department of Pediatrics, Kobe University Graduate School of Medicine, Kobe 650-0017, Japan; awahiro@med.kobe-u.ac.jp (H.A.); mmatsu@med.kobe-u.ac.jp (M.M.); natsu@med.kobe-u.ac.jp (M.N.); 3 Biomedical Analysis and Pathology Research Group, Discovery Science and Technology Department, Daiichi Sankyo RD Novare Co., Tokyo 134-8630, Japan; kawaguchi.tatsuya.sg@rdn.daiichisankyo.co.jp; 4 Department of Community Medicine and Social Healthcare Sciences, Kobe University Graduate School of Medicine, Kobe 650-0017, Japan; nishio@lion.kobe-u.ac.jp

**Keywords:** dystrophin, isoform, Dp116, Dp118, Duchenne muscular dystrophy, Schwann cell

## Abstract

The Duchenne muscular dystrophy (*DMD*) gene is one of the largest genes in the human genome. The gene exhibits a complex arrangement of seven alternative promoters, which drive the expression of three full length and four shorter isoforms. Dp116, the second smallest product of the *DMD* gene, is a Schwann cell-specific isoform encoded by a transcript corresponding to *DMD* exons 56–79, starting from a promoter/exon S1 within intron 55. The physiological roles of Dp116 are poorly understood, because of its extensive homology with other isoforms and its expression in specific tissues. This review summarizes studies on Dp116, focusing on clinical findings and alternative activation of the upstream translation initiation codon that is predicted to produce Dp118.

## 1. Introduction

The Duchenne muscular dystrophy (*DMD*) gene is one of the largest genes in the human genome, spanning over 2400 kb on the X-chromosome. The gene is comprised of 79 exons, which encode dystrophin, a 427-kDa protein of 3685 amino acid residues. Mutations in the *DMD* gene result in dystrophin deficiency, which causes Duchenne muscular dystrophy (DMD), the most common inherited muscle disease in childhood. DMD is characterized by fatal progressive muscle wasting. The cardinal symptom of DMD is muscle weakness, which first appears at age 4–6 years and becomes more severe over time. Cardiac or respiratory complications are life-threatening. DMD may also be accompanied by non-muscle symptoms [1], including mental retardation [2], red–green color vision impairment [3], electroretinogram abnormalities [4] and short stature [5,6].

The *DMD* gene exhibits a highly complex arrangement of seven alternative promoters, which drive the expression of three full length Dp427 isoforms, Dp427c, Dp427m and Dp427p, and four shorter isoforms, Dp260, Dp140, Dp116 and Dp71 [7]. Although the expression of Dp71 is ubiquitous in cells, the expression of other dystrophin isoforms is tissue-specific, with Dp427m expressed in skeletal and cardiac muscles, Dp427c in neurons of the cortex, Dp427p in cerebellar Purkinje neurons, Dp260 in retina, Dp140 in brain and kidney tissues, and Dp116 in Schwann cells [7]. Dp260 deficiency is associated with electroretinogram abnormalities [8,9]; Dp140 is involved in cerebral development and blood flow regulation [10,11]; and Dp71 has multiple physiological roles in cellular processes, including cell adhesion and cell division [12].

Dp116 is the second smallest product of the *DMD* gene and is encoded by a transcript corresponding to *DMD* exons 56–79, starting from a promoter/exon S1 within intron 55 [13]. The involvement of Dp116 in non-muscle symptoms of DMD remains unclear. This review summarizes studies of human Dp116, focusing on clinical findings and alternative activation of the upstream translation initiation codon to produce Dp118.

## 2. The Structure of Dp116 cDNA and Its Relationship to the *DMD* Gene

**Figure 1:**
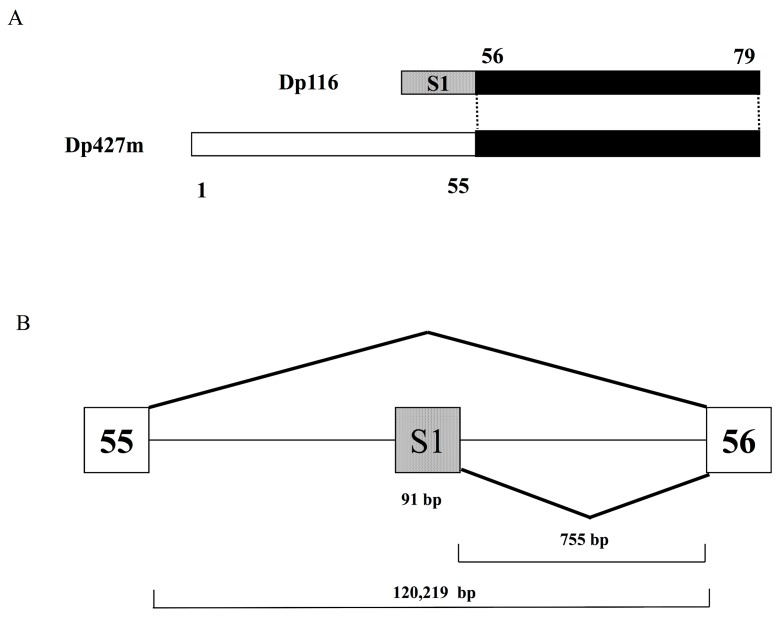
Dp116 cDNA and gene structures (**A**) Structure of Dp116 cDNA schematic depiction of the exon content of Dp116 cDNA (top) and Dp427m (bottom). Bars represent exons and numbers over and under bars indicate exon numbers. Dotted bars indicate the first exon of Dp116 (S1) and filled bars indicate the exons common to Dp116 and Dp427m. (**B**) Genomic structure of the exon S1 region schematic diagram showing the genomic region encompassing exon S1. Open and shaded boxes represent Duchenne muscular dystrophy (*DMD*) exons and the first exon of Dp116 (exon S1), respectively. Lines indicate introns. Diagonal lines indicate splicing patterns.

Dp116, also known as S-dystrophin or apodystrophin-2 [14], was the fifth isoform of dystrophin to be identified, after Dp427m, Dp427c, Dp427p and Dp71, and the second isoform found to have a promoter in downstream *DMD* introns [13]. Cloning of the full length Dp116 transcript from human sciatic nerve RNA showed that Dp116 cDNA consisted of specific exon S1 and *DMD* exons 56–79 (Figure 1A). Exon S1 is 91 bp long and is located in intron 55, more than 100 Kb downstream of *DMD* exon 55 and 755 bp upstream of exon 56 (Figure 1B). The splicing of exons S1 and exon 56 generates the full-length Dp116 transcript [13]. The promoter of Dp116 has not been well characterized. Because Dp116 is primarily expressed in the peripheral and central nervous systems [13], the promoter is likely under the control of neuron-specific regulators.

## 3. Characteristics of Dp116 Protein

Dp116 is an alternative C-terminal short isoform of Dp427m. The latter includes four domains: an N-terminal actin-binding domain, a central rod-like domain consisting of 24 spectrin-like triple helical repeats, a cysteine-rich domain and a C-terminal domain. The cysteine-rich domain of Dp427m contains areas for interactions with neuronal nitric oxide synthase, β-dystroglycan, syntrophins and dystrobrevins. Dp427m forms the dystrophin-associated protein complex (DAPC), which bridges the inner cytoskeleton and the extracellular matrix.

**Figure 2:**
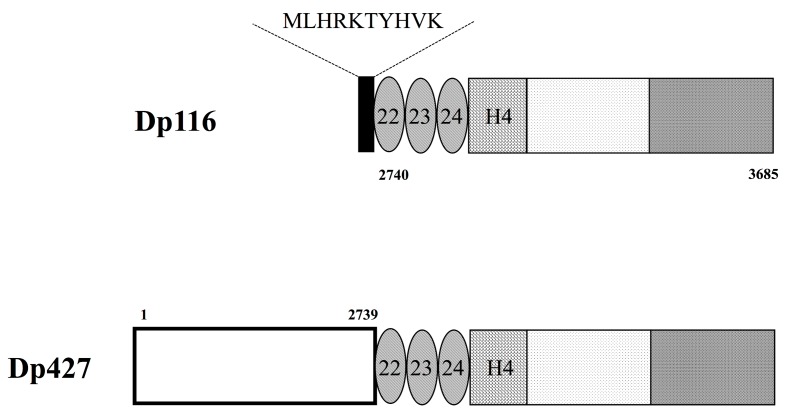
Structure of Dp116 protein. Schematic diagram showing the structure of Dp116 protein. The 10 N-terminal, Dp116-specific amino acids (black box) are present upstream of three spectrin-like repeats (oval), one hinge (striped box), and cysteine-rich and C-terminal domains (dotted and shaded boxes, respectively). Dp116 contains amino acid residues 2740–3685 of Dp427m, with the former also containing a long amino-terminal sequence consisting of 2739 amino acid residues (open box).

Full length Dp116 consists of 956 amino acids with a calculated molecular weight of 109,811 daltons (Accession No. NM_004014.2). Dp116 is characterized by its first exon (exon S1), which encodes 10 amino acids, MLHRKTYHVK. This unique N-terminal sequence does not correspond to any sequence within the first 2739 amino acids of Dp427m (Figure 2). However, the remaining 946 amino acids of Dp116 are identical to amino acid residues 2740–3685 of Dp427m. Structurally, Dp116 consists of three spectrin-like repeats, one hinge region, and the cysteine-rich and C-terminal domains of Dp427m, but lacks the N-terminal actin-binding domain and the central rod-like domain consisting of 21 spectrin-like repeats of Dp427m (Figure 2). Similar to Dp427m, Dp116 can form DAPCs. The roles of the 10 N-terminal amino acids of Dp116, MLHRKTYHVK, remain unclear.

## 4. Characterization of Dp116

Analyses of Dp116 mRNA and protein are hampered by their high degree of homology to other dystrophin isoforms and their expression limited to Schwann cells. The roles of Dp116 in humans may be extrapolated from its properties in other species or from the expression in humans of Up113, an autosomal homologue of Dp116.

### 4.1. Non-Human Studies of Dp116

The expression of Dp116 was assessed in the peripheral nerves and spinal cord of monkeys [15], and in glia, but not neurons, of the avian parasympathetic ciliary ganglion [16]. In rat sciatic nerve, Dp116 was expressed in a thin rim surrounding each Schwann cell-axon unit [17], whereas, in rabbit sciatic nerve, Dp116 was found to co-localize with β-dystroglycan in the sheath around each separate Schwann cell-axon unit [18,19]. In hamster peripheral nerve, Dp116 was shown to be a component of DAPC and to be involved in the stabilization of myelin [20].

**Figure 3:**
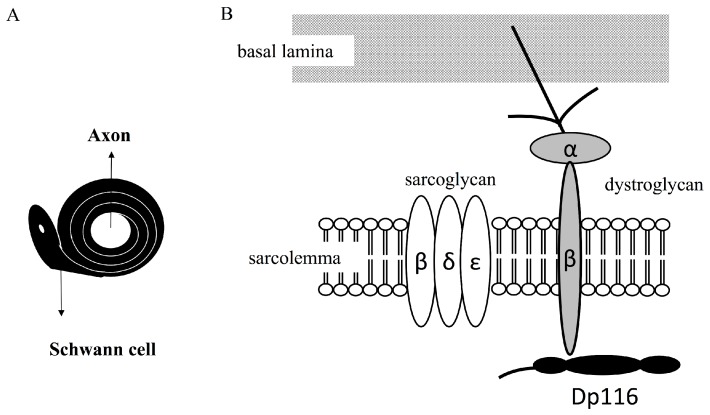
Dystrophin-associated protein complex (DAPC) formed by Dp116 in myelinating Schwann cells. Cross section of an axon surrounded by a Schwann cell (**A**). Schematic diagram showing a DAPC formed by Dp116 (**B**). Dp116 (black) interacts with beta-dystroglycan (gray), which is linked to basement membrane through alpha-dystroglycan (light gray) and laminin. Dp116 interacts with dystrophin associated proteins, such as epsilon, gamma, and beta sarcoglycans.

Dp116 was also found to be a component of DAPC in Schwann cell membranes of normal and *mdx* mice, a murine model of DMD [21,22]. Dp116 expressed in Schwann cells forms DAPC, as does Dp427m [23] (Figure 3). A complex in mouse Schwann cells containing Dp116 was found linked to dystrophin-related protein 2 (Drp2)/periaxin complexes, with a loss of Drp2 resulting in a corresponding increase in Dp116 [24]. During DAPC formation by Drp2, Dp116 was supposed to compensate Drp2 for the maintenance of DAPC in Cajal bands. The ATP-binding cassette transporter A1 (ABCA1) cholesterol transporter was shown to associate with Dp116/syntrophin complexes in Cajal bands of Schwann cells [25].

Interestingly, Dp116 mRNA and protein were found to be expressed in rat adipose tissue, with Dp116 protein forming DAPCs [26]. Moreover, Dp116 expression was observed in adult hearts in *Drosophila* [27], despite the lack of Dp116 expression in mouse cardiac muscle [13].

### 4.2. Up113, an Autosomal Homologue of Dp116

Utrophin is an autosomal homologue of dystrophin, suggesting that utrophin expression may compensate for dystrophin deficiency in the treatment of DMD [28]. In contrast to dystrophin, only one short isoform of utrophin has been well characterized to date. Up113, also called G-utrophin because it was initially detected in ganglia, is encoded by a 5.5-kb mRNA with a unique N-terminus, consisting of 48 amino acids, fused to a truncated spectrin-like repeat-20 domain, followed by cysteine-rich and C-terminal domains [29]. Up113 diverges from utrophin at the same point as does Dp116 from dystrophin. Therefore, Up113 is considered an autosomal homolog of Dp116. The N-terminal domains of Up113 and Dp116 both contain a consensus phosphorylation site for protein kinase C [29].

Up113 appears to be the predominant utrophin transcript in the brain and is specifically expressed in the adult mouse brain [29]. However, dystroglycan transcripts have not been detected in areas of Up113 expression, indicating that Up113 binds to an alternative functional equivalent of beta-dystroglycan [29]. Up113 in mouse brains localizes to limited locations, such as the thalamic reticular nucleus and hypothalamus, suggesting that its expression is selective [30].

### 4.3. Transgenic Expression of Human Dp116 in Mdx Mice

Transgenic expression of human Dp116 in *mdx* mice resulted in the preservation of functional muscle mass and an extended lifespan, without preventing dystrophy [31]. In contrast, Dp116 had no effect on dystrophic injury, as determined by muscle histopathology and serum creatine kinase levels. Dp116 also failed to restore normal fiber-type distribution or the post-synaptic architecture of the neuromuscular junction. These findings indicated that the DAPC formed by Dp116 is critical for the growth and maintenance of muscle mass, a function that is independent of the ability to prevent dystrophic pathophysiology [31].

## 5. Clinical Findings Associated with Dp116

DMD is a multiorgan disease, with complex varieties of signs and symptoms. Complications specifically associated with Dp116 deficiency remain unclear. Dp116 mRNA and protein expression is limited to human Schwann cells and fibroblasts [13,32]. Schwann cells localize along axons of the peripheral nervous system, suggesting that Dp116 deficient DMD may be characterized by motor and/or sensory neuron abnormalities. Although severe muscle damage in DMD may mask abnormalities in peripheral neurons, patients with DMD were shown to have abnormal tibial somatosensory evoked potentials [33]. Deficiency of Dp116 in peripheral nerve tissue may induce sensory impairment, inhibiting gastro-intestinal transport; however, gastro-intestinal symptoms have been underreported by some DMD patients with a variety of severe symptoms [34,35,36]. DMD patients with Dp116 deficiency did not show overt peripheral neuropathy [37,38,39,40]. In contrast, one patient with a splice site mutation in the *DMD* gene that abolished Dp116 expression was reported to have a demyelinating neuropathy [23]. These findings suggested the need to carefully examine Dp116-deficient patients with DMD.

About 7% of DMD patients are Dp116 deficient; thus, if Dp116 deficiency alone is directly responsible for the development of complications, the incidence of complications is about 7% [6]. A survey of complications in over 200 DMD patients found that the five most commonly reported conditions were cognitive deficits, constipation, anxiety problems, depression, and obesity [35]. Neuropsychiatric problems, such as attention-deficient/hyperactivity disorder (ADHD) and autism spectrum disorder, were also reported. Conditions with an incidence less than 7% included cancer, cerebral palsy, pseudo tumor cerebri, thrombosis, epilepsy, diabetes, gall stones and inflammatory bowel disease [35].

The prevalence of epilepsy has been reported higher in DMD patients (6.3%) than in the general pediatric population (0.5–1%) [41]. However, all DMD patients with epilepsy surveyed to date were found to have mutations outside the Dp116 coding region of the *DMD* gene, suggesting that Dp116 deficiency is unrelated to epilepsy.

As Dp116 deficient DMD patients also lack Dp427, Dp260 and Dp140, it is difficult to identify a Dp116 deficient-specific phenotype. To avoid complicated isoform deficiency, it is necessary to identify patients with mutations in exon S1. However, some patients with exon S1 mutations may not have DMD. No mutations located within exon S1 are listed in a large mutation data base [42]. Although X-linked mental retardation is a heterogeneous entity, one type of X-linked mental retardation has been linked to the *DMD* gene, although the location within this gene has not been determined [43]. These findings suggest that X-linked mental retardation may be associated with mutations in exon S1. Sequencing of the *DMD* gene in patients with X-linked mental retardation is required to confirm this hypothesis.

Cognitive impairment seems to correlate with Dp116 deficiency, as Dp116 is expressed in the brain. To date, however, cognitive impairment has been observed in patients with a deficiency of Dp140 or Dp71, but not of Dp116 [44,45,46,47].

Because Dp116 is expressed in fibroblasts, Dp116 abnormalities in patients with DMD, deranging the G-protein signaling pathway, have been associated with a hemorrhagic tendency during surgery [32]. Dp116 expression has been evaluated in patients with neuropathy complicated by diabetes mellitus. Immunolocalization of Dp116 in the sural nerve, however, did not differ in patients with diabetic neuropathy and normal controls [48]. Recently, an unfamiliar phenotype of temporomandibular disorder was shown linked with a single nucleotide polymorphism in the *DMD* gene [49]. But this needs further study to correlate with Dp116.

## 6. Dp118, a New Candidate Product from *DMD* Exon S1

### 6.1. Dp118 Produced by Activation of an Alternative Translation Initiation Site

**Figure 4:**
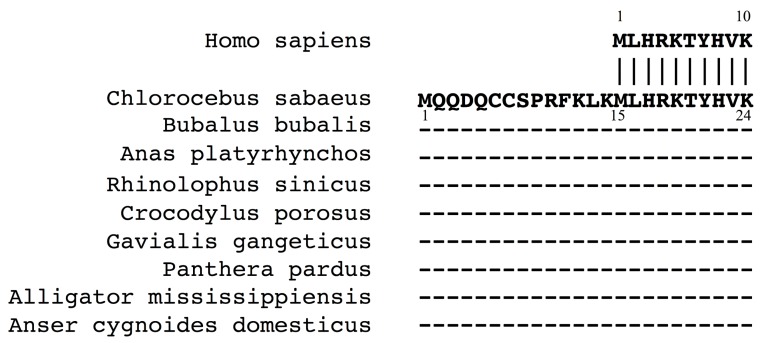
Proteins homologous to the 10 N-terminal amino acids of human Dp116. Blastp identification of proteins with N-terminal sequences completely identical to those of human Dp116 (top). The nine sequences from other animals were identical at amino acids 15–24. The remaining 14 amino acid residues in their N-termini were not homologous to human sequences.

Efforts using Blastp [50] to identify proteins with sequences homologous to 10 amino acids encoded by *DMD* exon S1 revealed 10 proteins with identical sequences (Figure 4). One was human Dp116, whereas the other nine had the identical amino acid sequences at the 15–24th amino acid residues. These products represented dystrophin isoforms from nine different animals, with all nine proteins having identical sequences at amino acids 1–24. This finding strongly suggested that the N-terminal end of human Dp116 is a truncated version.

**Figure 5:**
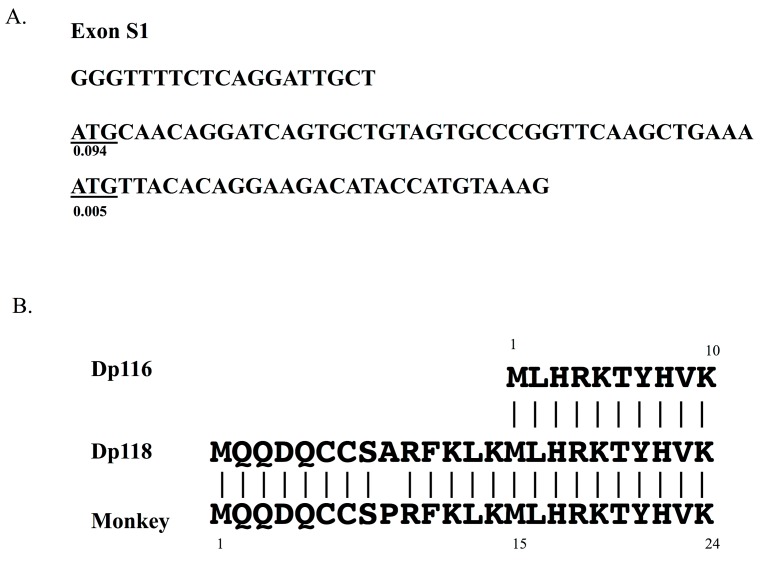
Dp118, an alternative translation initiation activation product of Dp116 transcript. (**A**) An alternative translation initiation site upstream of the known site. The 91 bp exon S1 sequences are shown. The ATG initiation codon is underlined, with this reading frame encoding 10 amino acids. Another ATG codon was identified 14 codons upstream of the authentic site. This upstream codon produces a protein 14 amino acids longer than Dp116, with the product named Dp118. Numbers under the ATG codons are the translation initiation score. (**B**) N-terminal amino acid sequence of Dp118. The 24 amino acids encoded by human and animal exons S1 are compared. Amino acid 9 differed, with alanine (A) present in humans, but proline (P) in monkeys. Dp116 is included for comparison.

The sequence located 5′ to the Dp116 translation start site was examined in human Dp116 cDNA. The ATG initiation codon in frame was identified as the fourteenth codon upstream of the known Dp116 translation start codon (Figure 5), suggesting that *DMD* exon S1 encodes 24, not 10, amino acids. The derived 24 amino acid sequence was MQQDQCCSARFKLKMLHRKTYHVK (underlined: Dp116). Translation of the first 14 amino acids, MQQDQCCSARFKLK, would increase the molecular weight of the product by nearly 2 kDa, yielding a protein product that would be called Dp118.

The possibility of Dp118 expression was further examined. Comparison of the 14 amino acid sequence in humans with the sequences of other species showed a difference in only one of these amino acids (Figure 5). That is, the ninth residue was alanine in human, but proline in other species. This high homology strongly indicated that Dp118 is expressed in humans. This single amino acid difference was found to result from a human-specific change from G to C in nucleotide 44 of exon S1.

The potential to act as a translation initiation site was also examined. The Kozak consensus sequence [51] was found to be CC(A/G)CCATG(G)(A/G represents A or G). Dp116 has the sequence TGAAAATGT, whereas Dp118 has the sequence TTGCTATGC (underlined: matched sequence), indicating that Dp118 had a greater similarity to the Kozak consensus sequence. Using DNA TIS miner [52], which can recognize translation initiation sites in DNA sequences [53], the scores for Dp118 and Dp116 were found to be 0.094 and 0.005, respectively. Because higher scores indicate a greater likelihood of being a true translation initiation signal, Dp118 is likely the real protein product of this transcript.

As two translation initiation codons direct the expression of two isoforms of annexin VI [54], two start codons in the Dp116 transcript may result in the production of both Dp116 and Dp118. Western blot analysis of mouse trigeminal and sciatic nerves detected doublet bands in the region of 116 kDa [13]. In silico analysis found that additional 14 amino acids in human Dp116 [55]. Therefore, alternative activation of translation initiation codons is highly possible in Dp116 transcripts.

### 6.2. Characterization of Dp118

To understand the difference between Dp116 and Dp118, the 14 additional amino acids, MQQDQCCSARFKLK, were examined. This sequence contained two cysteine residues, amino acids that often play essential roles in protein structure and function. Cysteines can stabilize protein structure through disulfide bond formation, can maintain proper maturation and localization through protein–protein intermolecular interactions, or provide thiol groups for reactions with molecular substrates. Intramolecular disulfide bond formation, favored in oxidizing extracellular and endoplasmic reticulum compartments, provides structural support for native function and localization, with loss of disulfide bonds resulting in mistargeting or malfunction of receptors and transporters [56,57]. Therefore, the C residues in Dp118 may be involved in the maintenance of its structure.

The four amino acid sequence SPRF in Dp116 of other animals is a potential phosphorylation site for protein kinase C [29]. In humans, this sequence was SARF, making human Dp118 different from Dp116 of other animals.

### 6.3. Future Studies to Confirm the Presence of Dp118

Although findings suggest that Dp118 is expressed in humans, confirmation is required by assaying the protein product. This, in turn, requires the production of antibodies specific to the 14 amino acid sequence, MQQDQCCSARFKLK, and immunostaining of Schwann cells with these antibodies. Alternatively, it is necessary to detect a peptide corresponding to these 14 amino acids by MS/MS analysis of Schwann cell extracts. If Dp118 expression is confirmed, it is necessary to repeat experiments utilizing transgenic mice expressing human Dp118 [31,58], in as many as these studies utilized transgenic mice expressing Dp116, not Dp118.

## 7. Conclusions

Dp116 is a dystrophin isoform specifically expressed in Schwann cells and forming DAPC in membranes. The clinical phenotype of Dp116 deficiency remains to be clarified. Dp118, an alternative translation initiation product, may be expressed in humans.
